# Intrapersonal Positive Future Thinking Predicts Repeat Suicide Attempts in Hospital-Treated Suicide Attempters

**DOI:** 10.1037/a0037846

**Published:** 2014-09-01

**Authors:** Rory C. O’Connor, Roger Smyth, J. Mark G. Williams

**Affiliations:** 1 Suicidal Behavior Research Laboratory, Institute of Health & Wellbeing, University of Glasgow; 2 Department of Psychological Medicine, Royal Infirmary of Edinburgh, Edinburgh, Scotland; 3 Department of Psychiatry, University of Oxford

**Keywords:** suicidal, psychology, prospective, cognitive

## Abstract

***Objective:*** Although there is clear evidence that low levels of positive future thinking (anticipation of positive experiences in the future) and hopelessness are associated with suicide risk, the relationship between the content of positive future thinking and suicidal behavior has yet to be investigated. This is the first study to determine whether the positive future thinking–suicide attempt relationship varies as a function of the content of the thoughts and whether positive future thinking predicts suicide attempts over time. ***Method:*** A total of 388 patients hospitalized following a suicide attempt completed a range of clinical and psychological measures (depression, hopelessness, suicidal ideation, suicidal intent and positive future thinking). Fifteen months later, a nationally linked database was used to determine who had been hospitalized again after a suicide attempt. ***Results:*** During follow-up, 25.6% of linked participants were readmitted to hospital following a suicide attempt. In univariate logistic regression analyses, previous suicide attempts, suicidal ideation, hopelessness, and depression—as well as low levels of achievement, low levels of financial positive future thoughts, and high levels of intrapersonal (thoughts about the individual and no one else) positive future thoughts predicted repeat suicide attempts. However, only previous suicide attempts, suicidal ideation, and high levels of intrapersonal positive future thinking were significant predictors in multivariate analyses. ***Discussion:*** Positive future thinking has predictive utility over time; however, the content of the thinking affects the direction and strength of the positive future thinking–suicidal behavior relationship. Future research is required to understand the mechanisms that link high levels of intrapersonal positive future thinking to suicide risk and how intrapersonal thinking should be targeted in treatment interventions.

## Abstract (public health significance statement toc summary)

This study highlights the importance of positive future thinking as a predictor of future suicidal behavior. Clinicians ought to consider the content of positive future thinking, as not all types of positive future thinking are protective over time.

Suicide and attempted suicide are major public health concerns, with approximately one million people dying by suicide annually across the globe (World Health Organization, n.d.). Indeed, as previous suicidal behavior is one of the strongest predictors of suicide (Hawton & van Heeringen, 2009), considerable research effort has been directed at understanding the etiology and course of suicide attempts. In recent years, there has also been increased recognition that we need to move beyond psychiatric categories and epidemiological risk factors to identify more specific markers of suicide risk (O’Connor & Nock, 2014; O’Connor, Smyth, Ferguson, Ryan, & Williams, 2013; van Heeringen, 2001). This has led to a concerted focus on basic science approaches to advance understanding of the psychological mechanisms that lead to suicidal behavior (e.g., Joiner, 2005; Nock et al., 2010; O’Connor, 2011; Van Orden et al., 2010; Williams, Barnhofer, Crane, & Beck, 2005).

One of the key advances has been the establishment of the link between hopelessness, defined as pessimism for the future, and suicide risk (O’Connor, Connery, & Cheyne, 2000; Petrie, Chamberlain, & Clarke, 1988; Beck, Steer, Kovacs, & Garrison, 1985). Hopelessness consistently predicts suicidal ideation and behavior (e.g., Brezo, Paris, & Turecki, 2006; Hawton, Saunders, & O’Connor, 2012). Although this bivariate relationship is robust, the work of MacLeod and others has demonstrated that hopelessness characterized by low levels of positive future thinking, rather than the preponderance of negative future thinking, is particularly important in the suicidal process (Hunter & O’Connor, 2003; MacLeod, Pankhania, Lee, & Mitchell, 1997; MacLeod et al., 1998; O’Connor, Fraser, Whyte, MacHale, & Masterton, 2008). Positive future thinking, defined as anticipation of positive experiences in the future, is usually assessed via the future thinking task (MacLeod et al., 1997), during which participants are asked to generate as many future events or experiences as possible that they are looking forward to.

Evidence from both clinical and nonclinical populations and from different research groups is consistent: Low levels of positive future thinking (i.e., few positive future thoughts) are associated with suicidality independent of depression, verbal fluency, and negative attributional style (Hunter & O’Connor, 2003; MacLeod et al., 1997; O’Connor, Connery, & Cheyne, 2000; Williams, Van der Does, Barnhofer, Crane, & Segal, 2008). This finding is clinically important, as positive future thinking provides targets for treatment intervention; theoretically, it is noteworthy as future plans and goals are key components of predominant models of suicidal behavior (O’Connor, 2011; Williams, 2001) as well as self-regulatory theories of wellbeing (Carver & Scheier, 1998).

Despite the accumulation of evidence in support of the positive future thinking–suicidality relationship, there are a number of key questions about the nature of this relationship that have yet to be addressed. First, does positive future thinking predict suicide-related outcomes over the medium to long term? To date, there is no evidence that low levels of positive future thinking have predictive validity beyond the first 2 to 3 months following an index suicide attempt. In the only clinical study of its kind, O’Connor et al. (2008) found that low levels of positive future thinking were better predictors of suicidal ideation than global hopelessness 2 to 3 months following a suicide attempt. To our knowledge, no other longer term studies of suicidal individuals have been conducted, and no previous study has investigated whether positive future thinking predicts actual suicidal behavior over time.

Second, it is unclear whether all types of positive future thinking are protective against suicidal behavior. The studies thus far have focused on establishing the presence of a relationship between the frequency of positive future thinking or the likelihood of these future events occurring and suicidality. None of the previous studies had been set up to investigate whether the *content* of positive future thinking affects the relationship between positive future thinking and suicidality. It is reasonable to posit, for example, that positive future thinking focused on changing a personal attribute (for the better) may not be protective if it is not possible to realize this change over time. Arguably, trait-like intrapersonal characteristics (e.g., being more confident, optimistic) may fall into this category. It may be, therefore, that high levels of such thinking are problematic in some circumstances. According to the integrated motivational–volitional model of suicidal behavior (IMV; O’Connor, 2011), such thinking, if experienced contemporaneously with feelings of entrapment (defined as the inability to escape from defeating or stressful circumstances, Gilbert & Allan, 1998; Williams, 2001), would increase the likelihood of suicidal thoughts developing. It is the thwarted motivation to escape that distinguishes entrapment from hopelessness, and it is posited that as entrapment increases (and no solutions are found) the likelihood that suicide will be considered as an escape strategy also increases (Gilbert & Allan, 1998; O’Connor et al., 2013; Taylor, Gooding, Wood, & Tarrier, 2011).

To address the former question directly, we modified an existing coding frame for positive future thinking (Godley, Tchanturia, MacLeod, & Schmidt, 2001) and classified the content of positive future thinking from a large sample of suicide attempters into seven categories. Using linkage methodology, we were able to investigate (a) whether positive future thinking predicts repeat suicidal behavior up to 15 months following an index suicide attempt (beyond the effects of traditional clinical risk factors), and (b) whether the content of positive future thinking affects the relationship between positive future thinking and repeat suicidal behavior.

## Method

### Participants and Procedure

We recruited 388 patients who were seen by the liaison psychiatry service the morning after presenting at a single general hospital in Edinburgh, Scotland, following a suicide attempt between January 2008 and September 2009. The hospital provides a full range of acute medical and surgical services, including an accident and emergency service. The vast majority of patients had presented with overdose (93%, *n* = 361). Exclusions were limited to participants who were unfit to participate (e.g., actively psychotic), who were unable to give informed consent (e.g., medically unfit to give informed consent), who were participating in one of the other studies being conducted in the hospital, or who were unable to understand English. Approximately 10% of participants who were approached declined to take part (10.2%, *N* = 44). There were 220 females and 168 males, with an overall mean age of 35.3 years (*SD* = 13.91, range = 16–71 years). The men (*M* = 38.40, *SD* = 14.04) and women (*M* = 32.92, *SD* = 13.36) did not differ significantly in age, *t*(386) = 3.92, *ns*. Ethnicity was not recorded.

Baseline data were collected in hospital, usually within 24 hr of admission. The Information Services Division of the National Health Service Scotland maintains a national database of hospital records and mortality data. This nationally linked database is a powerful resource, as it allows us to determine whether a patient is readmitted to hospital in Scotland with self-harm at any time since their index episode. We asked the Information Services Division to extract hospital admissions for self-harm in the period between the index self-harm episode and 15 months later for each patient. We also reviewed the electronic medical records of those patients who were hospitalized again following self-harm during the follow-up period to determine whether the repeat self-harm episode was a suicide attempt or not.

Participants completed the following measures in hospital.

### Baseline Measures

#### Positive future thinking

Positive future thinking was recorded via the future thinking task (MacLeod et al., 1997). This requires participants to think of potential future experiences that they are looking forward to across three time periods: the next week (including today), the next year, and the next 5 to 10 years. On each occasion, participants have 1 min to think of future experiences for a given time period; this is repeated until all three periods are assessed. Before administration of the future thinking task, all participants complete the standard verbal fluency task (to control for general cognitive fluency) in which they have to generate as many words as possible to three letters (*F, A, S*), with 1 min allowed per letter. Consistent with previous research (MacLeod et al., 1997), the time periods are aggregated to yield total positive future thinking scores (i.e., the total number of positive future thoughts per participant). The contents of positive future thinking were coded according to a modified version of Godley et al.’s (2001) coding frame for positive future thinking to yield a total number of positive future thoughts per category (see Table 1). There were seven categories of positive future thinking. *Social/interpersonal* relates to positive future thinking that involves at least one other person (e.g., marriage). *Achievement* relates to the anticipation of any achievement-related event (e.g., new job). *Intrapersonal* thinking is any thought that concerns the individual and no-one else (e.g., being happy). *Leisure/pleasure* refers to any event or activity that is undertaken for leisure or pleasure (e.g., going on holiday). *Health of others* (e.g., mother getting better) and *financial and home* (e.g., decorating the house) describe thinking that concerns improvement in the health of family or friends and any aspect of finance or home, respectively. The final category, *other*, describes any thinking that does not fit into the preceding categories. Three raters independently rated 15% of the responses and agreement was good (κ = .83, .90, .85 for raters 1 + 2, 1 + 3, and 2 + 3, respectively). All of the responses were then categorized by the first coder.[Table-anchor tbl1]

#### Depression

The Beck Depression Inventory (Beck, Steer, & Brown, 1996) is a well-established measure of depressive symptomatology. It consists of 21 groups of statements that assess the presence of depressive symptoms in the past 2 weeks with good reliability and validity. Cronbach’s α was .91.

#### Hopelessness

Hopelessness was measured using the 20-item Beck Hopelessness Scale. This is reliable and valid and has been shown to predict eventual suicide (Beck, Schuyler, & Herman, 1974; Beck, Steer, Kovacs, & Garrison, 1985). In the present study, internal consistency was very good (Kuder-Richardson–20 = .92).

#### Suicidal ideation

Participants’ thoughts of suicide over the past week were assessed via the 21-item Scale for Suicide Ideation (SSI; Beck, Steer, & Ranieri, 1988; Beck, Steer, & Brown, 1996). Cronbach’s α was .94.

#### Suicide intent

Suicide intent was assessed via the SSI (Beck et al., 1974). The SSI consists of 15 items that assess the objective circumstances related to a suicide attempt (eight items) and self-reported beliefs about one’s intention (seven items). Cronbach’s α was .72.

### Outcome Measure

#### Readmission to hospital with a suicide attempt

An episode of self-harm was recorded if a patient was admitted to any hospital in Scotland with self-harm in the 15 months following the index episode. For this data set, the Information Services Division successfully linked 96.4% of the sample (*n* = 374/388). Where a patient was readmitted to hospital with self-harm during the study period, we reviewed their medical records to ascertain whether this episode was a suicide attempt or not. We were able to determine the presence/absence of suicidal intent in 93.1% (94/101) of those who were admitted to hospital with self-harm again during the study period. Therefore, all analyses are based on the 367 participants who were linked and for whom we have suicide intent data if they were readmitted to hospital with self-harm (which represents 95% of the original sample). Two trained coders independently rated the extracts from the medical records and agreed on all cases. Coders were unaware of any of the baseline measures.

#### Statistical analyses

We conducted a series of univariate logistic regression analyses for each predictor of a future suicide attempt. The total number of positive future thoughts per category is entered into the regression analyses. Although we are interested specifically in the positive future thinking logistic regression analyses, we present the findings for other established predictors of suicidal behavior (i.e., depression, hopelessness, suicide ideation, past suicide attempts). To test the two hypotheses, we also conducted multivariate logistic regression analyses including all significant univariate predictors, as appropriate.

## Results

### Linked Sample

There were 208 women and 159 men with an overall mean age of 35 years (*SD* = 13.7, range: 16–71 years) in the linked sample. At baseline, 39.0% of participants (*n* = 143) reported no previous suicide attempts, 24.0% of participants reported one previous attempt (*n* = 88), 8.7% reported two previous attempts (*n* = 32) and 28.3% reported three or more previous episodes (*n* = 104). As anticipated, all indices of psychological distress were positively correlated (see Table 2). For the most part, the different categories of positive future thinking were negatively correlated with depression, hopelessness, and suicidal ideation. Suicidal intent was negatively correlated with two of the positive future thinking categories (interpersonal and achievement positive future thinking), as well as positively correlated with the psychological distress indicators. Finally, more previous suicide attempts were associated with increased distress and less interpersonal, achievement and leisure/pleasure positive future thinking.[Table-anchor tbl2]

### Individual and Multivariate Predictors of Repeat Suicide Attempts

Between Time 1 and Time 2 (15 months after the index episode), 25.6% (*n* = 94) of the linked participants either were readmitted to hospital with a suicide attempt or died by suicide (5/94). We conducted a series of logistic regression analyses to determine the variables for entry into the multivariate analyses. Established correlates of suicidal behavior (e.g., depression, suicidal ideation) were included in the analyses to ensure a robust test of the positive future thinking–repeat suicide attempt relationship. None of the demographic variables were significant univariate predictors of repeat suicide attempts (see Table 3). However, among the clinical predictors, the number of previous suicide attempts, suicidal ideation, hopelessness, and depression emerged as significant predictors. In respect to positive future thinking,^1^ low levels of achievement and financial positive future thinking were associated with suicide attempts between Time 1 and Time 2, whereas high levels of intrapersonal positive future thinking was also significant (see Table 3).[Table-anchor tbl3]

To investigate whether positive future thinking has utility in predicting repeat suicidal behavior up to 15 months following an index suicide attempt (beyond the effects of traditional clinical risk factors) and whether the content of positive future thinking affects the relationship between positive future thinking and repeat suicidal behavior, the significant univariate predictors were entered into the multivariate logistic regression in two stages. The traditional clinical risk factors were entered at Step 1, followed by the positive future thinking variables at Step 2. As is evident in Table 4, intrapersonal positive future thinking is a significant predictor of repeat suicide attempts in the final model (*OR* = 1.25, 95% CI [1.07, 1.44]), and its inclusion adds incremental predictive validity over previous suicide attempts and suicidal ideation (χ^2^ = 11.34, *p* < .01).[Table-anchor tbl4]

## Discussion

The present study extends understanding of the relationship between positive future thinking and suicide attempts. First, the findings demonstrate that some intrapersonal positive future thoughts predict repeat suicidal behavior up to 15 months following an index suicide attempt. Second, they also show that the relationship between positive future thinking and suicidality varies as a function of the content of such thinking. Specifically, in the univariate analyses, high levels of intrapersonal positive future thinking were associated with the risk of repetition, whereas low levels of achievement and financial positive future thinking were associated with repeat suicidal behavior. What is more, the multivariate analyses suggest that intrapersonal positive future thinking is most pernicious of all, as the effects of achievement and financial future thinking were rendered nonsignificant when considered alongside past suicidal behavior, suicidal ideation, hopelessness, and depression.

The findings are also noteworthy because they highlight not only that the types of positive future thinking have differential predictive validity but crucially because they show that high levels of positive future thinking are not always protective. On the face of it, this may seem counterintuitive, given the generally accepted view that high levels of positive thinking buffer against distress in the face of life stress (e.g., O’Connor, O’Connor, O’Connor, Smallwood, & Miles, 2004). Moreover, closer inspection of the baseline correlations shows that the degree of protection also changes as a function of the individual’s current context. When participants are in crisis, in the hours following a suicide attempt, high levels of intrapersonal positive future thinking appear to be protective, as illustrated by the negative correlations between intrapersonal future thinking, hopelessness, and suicidal ideation. These baseline findings are consonant with the extant literature on positive future thinking, which has consistently demonstrated that suicidal individuals generate lower levels of positive future thinking than controls (e.g., Hunter & O’Connor, 2003; MacLeod et al., 1997).^2^

However, over the subsequent 15 months, the reverse relationship is apparent. The likelihood of another suicide attempt was elevated among those who reported more intrapersonal positive future thinking at baseline (when in crisis). As noted in the introduction, one possible explanation for the latter relationship may be that, over time, participants develop beliefs that their intrapersonal future thoughts are not attainable as they have not been able to achieve what they had expected within the intrapersonal domain over the duration of the study. It may be that these beliefs exacerbate their sense of entrapment, thereby increasing their risk of repeat suicidal behavior. Alternatively, it may simply be that the generation of positive future thinking is confounded by contemporaneous mood effects. The latter is unlikely, however, as baseline mood was controlled for in the multivariate analyses. Nonetheless, the unachievability hypothesis requires closer scrutiny in future research, as entrapment was not assessed in the present study, and assessing the impact of mood on intrapersonal versus external positive future thinking requires a specific test in which mood is experimentally manipulated. A further competing hypothesis is that frequent swings in self-image from high to low and vice versa that characterize some clients’ cognitions (e.g., clients with borderline personality or bipolar disorder) may account for the present findings. As we only assessed positive future thinking at one time point (and we also did not assess clinical disorder), it was not possible to test this hypothesis directly. Therefore, future research should investigate whether this instability in cognition has explanatory power in the present context.

Two other methodological points also merit comment. The first point relates to the test–retest reliability of the positive future thinking task. To our knowledge, this has not been formally tested; however, evidence from a recent experimental study in which positive future thinking was assessed twice within a single testing session suggests that responses are stable in the very short term (O’Connor & Williams, 2014). However, it is important to investigate this issue further to tease out whether, for example, intrapersonal positive future thinking is highly unstable when assessed over a period of days and weeks rather than hours.

Another issue relates to the extent to which the positive future thinking task is useful outside of the 24 hr following a suicide attempt. Although most studies have administered it within this time frame, other studies have employed it within 7 days of a suicidal episode (MacLeod et al., 2005), and others still have employed it in healthy populations (O’Connor & Williams, 2014; Williams et al., 2008) and found the expected relationships with hopelessness and suicidal ideation. Given this evidence, we do not think that the findings reported here are circumscribed to the immediate post-suicide-attempt period. Indeed, it is likely that the pattern of positive future thinking found in the perisuicidal period is similar to that found in the post-suicide-attempt period, but this is an empirical question. Indeed, it is critical that future research explore the trajectory of positive future thinking over time, to better understand the dynamic relationship between the levels of positive future thinking and suicide risk before, during, and after crisis.

### Implications

Irrespective of the mechanism(s) of effect, the preeminence of intrapersonal rather than other dimensions of positive future thinking, including interpersonal thoughts, is clear from the present findings, as the former was the only category of positive future thinking to emerge from the multivariate analyses. This pattern of findings is also consistent with the integrated motivational–volitional model of suicidal behavior (O’Connor, 2011), which argues that positive future thinking may increase the likelihood that suicidality emerges from entrapment beliefs. Furthermore, the present findings have implications for how to intervene effectively with those who have attempted suicide to reduce risk of repetition. They clearly suggest that the content of positive future thinking requires careful consideration as part of the formulation process. Indeed, it may be helpful to monitor the achievability or otherwise of intrapersonal positive future thinking and to develop strategies to maximize the likelihood that the intrapersonal expectations are attainable. Patients may also benefit from help with problem solving when the expectations are not realized. Alternatively, in situations where the expectations are unrealistic or unattainable (e.g., O’Connor, O’Carroll, Ryan, & Smyth, 2012; Wrosch, 2010), working collaboratively with the individual to disengage from such future expectations in a safe manner and engage with new, more realistic positive future thinking may bear fruit.

There are also a number of research implications. First, positive future thinking is treated as a continuous variable in the present study. It would be useful, therefore, to investigate whether there is a critical threshold at which positive future thinking becomes especially deleterious, but this is likely best achieved by also assessing the perceived achievability of the positive future thinking. It would also be useful to investigate an individual’s certainty about a positive event occurring in the future and how this relates to risk (Sargalska, Miranda, & Marroquin, 2011). Second, for pragmatic reasons we employed cognitive assessment to record current psychological state rather than conducting a formal clinical assessment. It may be helpful in the future, therefore, to investigate whether the relationship between positive future thinking and suicide risk varies as a function of clinical diagnostic category. Third, whereas we coded the contents of individuals’ thinking post hoc, it would be interesting to ask participants to generate specific types of positive future thinking to determine whether there are different ways in which individuals rate their own thinking. More generally, the findings highlight the utility of focusing on psychological processes to identify more specific markers of suicide risk (O’Connor & Nock, 2014).

Although the longitudinal design and the use of an objective outcome measure are notable strengths of the present study, there are a number of potential limitations that merit comment. First, as we concentrated on hospital admissions and mortality, the present study was not designed to capture less medically serious suicide attempts that did not come to the attention of clinical services. In addition, although unlikely, we also would have missed any hospitalizations or deaths that occurred outside Scotland. Also, the national linkage methodology did not record those individuals who presented to the emergency department but were subsequently discharged without hospitalization. Consequently, future research is required to determine whether a similar pattern of findings would hold for non-medically serious suicide attempts. It would also be useful to look at nonsuicidal self-injury as another self-destructive outcome variable. Finally, as all of the participants had attempted suicide at baseline, it is unclear whether high levels of intrapersonal positive future thinking predict a first episode suicide attempt.

## Conclusions

This is the first study to investigate whether the content of positive future thinking predicts repeat suicidal behavior over the medium term. The findings demonstrate clearly that the content of the thoughts affects the direction and strength of the positive future thinking–suicidal behavior relationship. Whereas previous research had shown that low levels of positive future thinking are associated with suicidal behavior, the present study found that high levels of intrapersonal positive future thoughts predict repeat suicide attempts over time. Future research is required to understand the mechanisms that link intrapersonal positive future thinking to suicide risk and how intrapersonal positive future thinking should be targeted in treatment interventions.

## Figures and Tables

**Table 1 tbl1:** Coding System for the Content of Positive Future Thinking and Mean Number of Thoughts as a Function of a Suicide Attempt or Suicide During Follow-Up

Category	Description	Number of positive future thoughts	
		Suicide attempt during follow-up *M* (*SD*)	No suicide attempt during follow-up *M* (*SD*)
Social/interpersonal	Social/interpersonal items include seeing family/friends, interpersonal events like marriage, divorce/separation, having children. The thought should involve at least one other person.	1.31 (1.76)	1.55 (1.85)
Achievement	Academic, job-related, or other test-related achievements include passing exams, getting into university/college, new job/promotion. School-related items are also included here.	0.61 (.99)	0.91 (1.32)
Intrapersonal	Intrapersonal items relate to the individual and no one else. Own health-related items are included here. Examples include getting better, not being depressed, being happy, being healthy, recovering, being more confident, etc.	1.45 (1.93)	0.95 (1.50)
Leisure/pleasure	Activities or events that are undertaken for leisure or pleasure. Examples include sport, birthdays, holidays, watching TV, shopping, dinner, etc. These can be sociable but they are included here because no one else is mentioned, as they can be done alone.	0.79 (1.16)	1.10 (1.55)
Health of others	Items that relate to the health of other family or friends. They can include improvements in mental/physical health or general wellbeing.	0.04 (.20)	0.02 (.15)
Financial and home	Items related to any aspect of finance/money or home are included here. Examples include moving house, decorating home, debts being paid off, etc.	0.32 (.68)	0.53 (.81)
Other	Items that do not fit into the categories above or where there is doubt about the category for which an item is best suited.	0.17 (.52)	0.16 (.44)
*Note.* This coding system was adapted from Godley et al. (2001).			

**Table 2 tbl2:** Correlations, Means, and Standard Deviations for All of the Study Variables for All Participants

Variable	Previous suicide attempt	Depression	Hopelessness	Suicidal ideation	Suicide intent	Social/interpersonal PFT	Achievement PFT	Intrapersonal PFT	Leisure/pleasure PFT	Health of others PFT	Financial PFT	Other PFT
Previous suicide attempt												
Depression	.250***											
Hopelessness	.237***	.691***										
Suicidal ideation	.295***	.593***	.592***									
Suicide Intent	.031	.287***	.290***	.473***								
Social/interpersonal PFT	−.105*	−.214***	−.248***	−.262***	−.156**							
Achievement PFT	−.116*	−.227***	−.273***	−.202***	−.161**	.242***						
Intrapersonal PFT	.033	−.025	−.195***	−.126*	−.010	.155**	.105*					
Leisure/pleasure PFT	−.127*	−.266***	−.286***	−.203***	.017	.177**	.180**	.076				
Health of others PFT	−.049	−.025	−.002	−.107*	−.074	.093	−.018	.167**	−.025			
Financial PFT	−.054	−.254***	−.260***	−.204***	−.064	.387***	.289***	.091	.195***	.069		
Other PFT	.020	.036	−.048	.074	.012	.028	−.018	.175**	.004	−.023	.101	
*M* (*SD*)	1.26 (1.24)	39.93 (12.12)	13.01 (5.29)	18.91 (10.04)	28.91 (4.11)	1.49 (1.83)	.83 (1.25)	1.08 (1.63)	1.02 (1.46)	.03 (.16)	.48 (.79)	.16 (.46)
*Note.* PFT = positive future thinking.												
* *p* < .05. ** *p* < .01. *** *p* < .001.												

**Table 3 tbl3:** Univariate Associations Between Predictors and Suicide Attempts or Suicide Between Time 1 and Time 2

Variable	*N* (%)	% attempted suicide T1–T2	OR	95% CI	*p*
Gender					
Male	159 (43.3)	24.2			
Female	208 (56.7)	22.1	.827	0.52, 1.33	.430
Marital status					
Married/partner	70 (19.1)	17.1			
Single/other	297 (80.9)	27.6	1.84	0.94, 3.61	.074
Employment status					
In employment/training	143 (39.0)	21.0			
Not in employment/training	224 (61.0)	28.6	1.51	0.92, 2.47	.105
Age	*M*	*SD*			
No repeat suicide attempt (T1–T2)	34.19	13.59			
Repeat suicide attempt (T1–T2)	37.20	13.70	1.02	1.00, 1.03	.066
Previous suicide attempts					
No repeat suicide attempt (T1–T2)					
Repeat suicide attempt (T1–T2)			1.42	1.18, 1.72	**.0001**
Suicidal intent					
No repeat suicide attempt (T1–T2)	28.77	4.09			
Repeat suicide attempt (T1–T2)	29.30	4.16	1.03	0.97, 1.09	.284
Suicidal ideation					
No repeat suicide attempt (T1–T2)	17.71	10.30			
Repeat suicide attempt (T1–T2)	22.40	8.33	1.05	1.03, 1.08	**.0001**
Hopelessness					
No repeat suicide attempt (T1–T2)	12.66	5.50			
Repeat suicide attempt (T1–T2)	14.48	4.64	1.07	1.02, 1.12	**.005**
Depression					
No repeat suicide attempt (T1–T2)	38.65	12.54			
Repeat suicide attempt (T1–T2)	43.86	10.75	1.04	1.02, 1.06	**.0001**
Positive Future Thinking					
Social/interpersonal PFT					
No suicide attempt (T1–T2)	1.55	1.85			
Suicide attempt (T1–T2)	1.31	1.76	0.93	0.81, 1.06	.264
Achievement PFT					
No Suicide attempt (T1–T2)	0.91	1.32			
Suicide attempt (T1–T2)	0.61	0.99	0.79	0.63, 1.00	**.045**
Intrapersonal PFT					
No suicide attempt (T1–T2)	0.95	1.50			
Suicide attempt (T1–T2)	1.45	1.93	1.19	1.04, 1.36	**.013**
Leisure/Pleasure PFT					
No Suicide attempt (T1–T2)	1.10	1.55			
Suicide attempt (T1–T2)	0.79	1.16	0.85	0.70, 1.02	.081
Others’ health PFT					
No suicide attempt (T1–T2)	0.02	0.15			
Suicide attempt (T1–T2)	0.04	0.20	1.98	0.55, 7.17	.299
Financial PFT					
No suicide attempt (T1–T2)	0.53	0.81			
Suicide attempt (T1–T2)	0.32	0.68	0.67	0.47, 0.95	**.026**
Other PFT					
No suicide attempt (T1–T2)	0.16	0.44			
Suicide attempt (T1–T2)	0.17	0.52	1.04	0.63, 1.72	.870
*Note.* Bold indicates statistical significance at the conventional levels. PFT = positive future thinking.					

**Table 4 tbl4:** Multivariate Logistic Regression Analysis to Predict Suicide Attempts or Suicide Between Time 1 and Time 2

Variable	Step 1				Step 2			
	OR	95% CI	*p*	χ^2^	OR	95% CI	*p*	χ^2^
Previous suicide attempts	1.29	1.06, 1.58	**.011**		1.27	1.04, 1.56	**.020**	
Suicidal ideation	1.03	1.00, 1.07	.077		1.04	1.00, 1.07	**.050**	
Hopelessness	0.99	0.92, 1.06	.700		1.00	0.93, 1.07	.944	
Depression	1.03	1.00, 1.06	.102		1.02	0.98, 1.05	.345	
Intrapersonal positive future thinking					1.25	1.07, 1.44	**.004**	
Achievement positive future thinking					0.88	0.68, 1.13	.307	
Financial positive future thinking					0.77	0.53, 1.14	.190	
Step χ^2^				26.22***				11.34**
Model χ^2^								37.56***
*Note.* Bold indicates statistical significance at the conventional levels.								
** *p* < .01. *** *p* < .001.								

## Footnotes

1 As general verbal fluency did not predict repeat suicide attempts (*OR* = .98, 95% CI [0.94, 1.01]), it was not considered any further in the main analyses.

2 It is worth highlighting that none of the earlier studies analyzed positive future thinking as a function of thought content.

## References

[c1] Beck A. T., Schuyler D., & Herman I. (1974). Development of the suicidal intent scales In Beck A. T., Resnik H. L. P., & Lettieri D. J. (Eds.), The prediction of suicide (pp. 45–56). Philadelphia, PA: Charles Press.

[c2] Beck A. T., Steer R. A., & Brown G. K. (1996). Manual for the Beck Depression Inventory-II. San Antonio, TX: Psychological Corporation.

[c4] Beck A. T., Steer R. A., Kovacs M., & Garrison B. (1985). Hopelessness and eventual suicide: A 10-year prospective study of patients hospitalized with suicidal ideation. The American Journal of Psychiatry, 142, 559–563. 3985195 10.1176/ajp.142.5.559

[c5] Beck A. T., Steer R. A., & Ranieri W. (1988). Scale for Suicide Ideation: Psychometric properties of a self-report version. Journal of Clinical Psychology, 44, 499–505. doi:10.1002/1097-4679(198807)44:4<499::AID-JCLP2270440404>3.0.CO;2-6 3170753

[c6] Brezo J., Paris J., & Turecki G. (2006). Personality traits as correlates of suicidal ideation, suicide attempts, and suicide completions: A systematic review. Acta Psychiatrica Scandinavica, 113, 180–206. doi:10.1111/j.1600-0447.2005.00702.x 16466403

[c7] Carver C. S., & Scheier M. F. (1998). On the self-regulation of behavior. Cambridge, England: Cambridge University Press. doi:10.1017/CBO9781139174794

[c8] Gilbert P., & Allan S. (1998). The role of defeat and entrapment (arrested flight) in depression: An exploration of an evolutionary view. Psychological Medicine, 28, 585–598. doi:10.1017/S0033291798006710 9626715

[c9] Godley J., Tchanturia K., MacLeod A., & Schmidt U. (2001). Future-directed thinking in eating disorders. British Journal of Clinical Psychology, 40, 281–295. doi:10.1348/014466501163698 11593956

[c10] Hawton K., Saunders K. E. A., & O’Connor R. C. (2012). Self-harm and suicide in adolescents. The Lancet, 379, 2373–2382. doi:10.1016/S0140-6736(12)60322-5 22726518

[c11] Hawton K., & Van Heeringen K. (2009). Suicide. The Lancet, 373, 1372–1381. doi:10.1016/S0140-6736(09)60372-X 19376453

[c12] Hunter E. C., & O’Connor R. C. (2003). Hopelessness and future thinking in parasuicide: The role of perfectionism. British Journal of Clinical Psychology, 42, 355–365. doi:10.1348/014466503322528900 14633412

[c13] Joiner T. (2005). Why people die by suicide. Boston, MA: Harvard University Press.

[c14] MacLeod A. K., Pankhania B., Lee M., & Mitchell D. (1997). Parasuicide, depression and anticipation of positive and negative future experiences. Psychological Medicine, 27, 973–977. doi:10.1017/S003329179600459X 9234475

[c15] MacLeod A. K., Tata P., Evans K., Tyrer P., Schmidt U., Davidson K., & Thompson S. (2005). Hopelessness and positive and negative future thinking in parasuicide. British Journal of Clinical Psychology, 44, 495–504. doi:10.1348/014466505X35704 16368029

[c16] MacLeod A. K., Tata P., Evans K., Tyrer P., Schmidt U., Davidson K., . . . Catalan J. (1998). Recovery of positive future thinking with a high-risk parasuicide group: Results from a pilot randomised controlled trial. British Journal of Clinical Psychology, 37, 371–379. doi:10.1111/j.2044-8260.1998.tb01394.x 9856290

[c17] Nock M. K., Park J. M., Finn C. T., Deliberto T. L., Dour H. J., & Banaji M. R. (2010). Measuring the suicidal mind: Implicit cognition predicts suicidal behavior. Psychological Science, 21, 511–517. doi:10.1177/0956797610364762 20424092 PMC5258199

[c18] O’Connor R. C. (2011). Towards an integrated motivational-volitional model of suicidal behaviour In O’Connor R. C., Platt S., & Gordon J. (Eds.), International handbook of suicide prevention: Research, policy & practice (pp. 181–198). Chichester, England: Wiley-Blackwell. doi:10.1002/9781119998556.ch11

[c19] O’Connor R. C., Connery H., & Cheyne W. (2000). Hopelessness: The role of depression, future directed thinking and cognitive vulnerability. Psychology, Health & Medicine, 5, 155–161. doi:10.1080/713690188 29156959

[c20] O’Connor R. C., Fraser L., Whyte M. C., MacHale S., & Masterton G. (2008). A comparison of specific positive future expectancies and global hopelessness as predictors of suicidal ideation in a prospective study of repeat self-harmers. Journal of Affective Disorders, 110, 207–214. doi:10.1016/j.jad.2008.01.008 18262284

[c21] O’Connor R. C., & Nock M. K. (2014). The psychology of suicidal behavior. Lancet Psychiatry, 1, 73–85. doi:10.1016/S2215-0366(14)70222-6 26360404

[c22] O’Connor R. C., O’Carroll R. E., Ryan C., & Smyth R. (2012). Self-regulation of unattainable goals in suicide attempters: A 2 year prospective study. Journal of Affective Disorders, 142, 248–255. doi:10.1016/j.jad.2012.04.035 22980400

[c23] O’Connor R. C., O’Connor D. B., O’Connor S. M., Smallwood J. M. & Miles J. (2004). Hopelessness, stress and perfectionism: The moderating effects of future thinking. Cognition & Emotion, 18, 1099–1120. doi:10.1080/02699930441000067

[c24] O’Connor R. C., Smyth R., Ferguson E., Ryan C., & Williams J. M. G. (2013). Psychological processes and repeat suicidal behavior: A four-year prospective study. Journal of Consulting and Clinical Psychology, 81, 1137–1143. doi:10.1037/a0033751 23855989 PMC3933214

[c25] O’Connor R. C., & Williams J. M. G. (2014). The relationship between positive future thinking, brooding, defeat and entrapment. Personality and Individual Differences, 70, 29–34.

[c26] Petrie K., Chamberlain K., & Clarke D. (1988). Psychological predictors of future suicidal behaviour in hospitalized suicide attempters. British Journal of Clinical Psychology, 27, 247–257. doi:10.1111/j.2044-8260.1988.tb00781.x 3191304

[c27] Sargalska J., Miranda R., & Marroquin B. (2011). Being certain about an absence of the positive: Specificity in relation to hopelessness and suicidal ideation. International Journal of Cognitive Therapy, 4, 104–116. doi:10.1521/ijct.2011.4.1.104

[c101] Taylor P. J., Gooding P., Wood A. M., & Tarrier N. (2011). The role of defeat and entrapment in depression, anxiety and suicide. Psychological Bulletin, 137, 391–420. 21443319 10.1037/a0022935

[c28] Van Heeringen K. (2001). Towards a psychobiological model of the suicidal process In van Heeringen K. (Ed.), Understanding suicidal behaviour (pp. 136–159). Chichester, United Kingdom: Wiley.

[c29] Van Orden K. A., Witte T. K., Cukrowicz K. C., Braithwaite S., Selby E. A., & Joiner T. E. (2010). The interpersonal theory of suicide. Psychological Review, 117, 575–600. doi:10.1037/a0018697 20438238 PMC3130348

[c30] Williams J. M. G. (2001). The cry of pain. London, England: Penguin.

[c31] Williams J. M. G., Barnhofer T., Crane C., & Beck A. T. (2005). Problem-solving deteriorates following mood challenge in formerly depressed patients with a history of suicidal ideation. Journal of Abnormal Psychology, 114, 421–431. doi:10.1037/0021-843X.114.3.421 16117579

[c32] Williams J. M. G., Van der Does A. J. W., Barnhofer T., Crane C., & Segal Z. S. (2008). Cognitive reactivity, suicidal ideation and future fluency: Preliminary investigation of a differential activation theory of hopelessness/suicidality. Cognitive Therapy and Research, 32, 83–104. doi:10.1007/s10608-006-9105-y

[c33] World Health Organization (n.d.). Suicide prevention (SUPRE). Retrieved from http://www.who.int/mental_health/prevention/suicide/suicideprevent/en/

[c34] Wrosch C. (2010). Self-regulation of unattainable goals and pathways to quality of life In Folkman S. (Ed.), Oxford handbook of stress, health and coping (pp. 319–333). Oxford, England: Oxford University Press. doi:10.1093/oxfordhb/9780195375343.013.0016

